# Nerve growth factor and Tropomyosin receptor kinase A are increased in the gastric mucosa of patients with functional dyspepsia

**DOI:** 10.1186/s12876-019-1133-7

**Published:** 2019-12-19

**Authors:** Haitao Shi, Shanshan Zhu, Bin Qin, Lianli Wang, Juan Yang, Guolong Lu, Fei Dai

**Affiliations:** 1 Department of Gastroenterology, The Second Affiliated Hospital of Xi’an Jiaotong University, No. 157 Xiwu Road, Xi’an, 710004 Shaanxi China; 2 Department of Infectious Diseases, Xi’an Central Hospital, Xi’an, 710001 Shaanxi China; 3 Department of Gastroenterology, Xi’an NO. 3 Hospital, Xi’an, 710021 Shaanxi China

**Keywords:** Functional dyspepsia, Nerve growth factor, Tropomyosin receptor kinase A, Enteric glial cells

## Abstract

**Background:**

Nerve growth factor (NGF) and enteric glial cells (EGCs) are associated with visceral hypersensitivity and gastrointestinal motility disorder, which may represent the pathogenesis of functional dyspepsia (FD). This study aimed to investigate the expression of NGF, its high affinity receptor tropomyosin receptor kinase A (TrkA) and the EGC activation marker glial fibrillary acidic protein (GFAP) in the gastric mucosa of patients with FD and the association of these proteins with dyspeptic symptoms.

**Methods:**

Gastric mucosal biopsies taken from 27 FD patients (9 epigastric pain syndrome (EPS) patients, 7 postprandial distress syndrome (PDS) patients and 11 EPS overlap PDS patients) and 26 control subjects were used for analysis. The expression of NGF, TrkA and GFAP was examined, and the association of these proteins with dyspeptic symptoms, including epigastric pain, postprandial fullness, early satiation and epigastric burning, was analysed.

**Results:**

The expression levels of NGF, TrkA, and GFAP in the gastric mucosa were significantly higher in the EPS group, the PDS group, and the EPS overlap PDS group than in the healthy control group. There was no significant difference between the FD subgroups. TrkA colocalized with GFAP, which indicated that TrkA was localized to EGCs, and the expression of TrkA in EGCs was significantly higher in the FD group than in the control group. Changes in the expression of NGF, TrkA, and GFAP were positively correlated with epigastric pain, postprandial fullness and early satiation but had no significant relationship with epigastric burning.

**Conclusions:**

The increased expression of gastric NGF, TrkA and GFAP might be involved in FD pathophysiology and symptom perception.

## Background

Functional dyspepsia (FD), a common functional gastrointestinal disease, is mainly manifested by bothersome epigastric pain, postprandial fullness, early satiation and epigastric burning [1]. However, there is a lack of organic, systematic or metabolic diseases that can explain the above symptoms. According to the Rome IV diagnostic criteria, FD can be divided into two subtypes, namely, postprandial distress syndrome (PDS) and epigastric pain syndrome (EPS) [2]. Clinically, PDS often overlaps with EPS. According to existing research, the worldwide incidence of FD is 10–30% [3]**.** The pathogenesis of FD remains unclear. According to the current research, it is mainly related to gastroduodenal dynamic abnormalities [4], visceral hypersensitivity [5], local environmental factors in the stomach, brain-gut axis, diet and other lifestyle factors.

The enteric nervous system (ENS) is composed of nerve components in the gastrointestinal tract and is similar to the central nervous system (CNS) in structure and function. It is an integral part of the autonomic nervous system. Enteric glial cells (EGCs) are major cellular components of myenteric and submucosal ganglia within the ENS and are distributed in the gastrointestinal muscle layer, submucosa and lamina propria [6]. In addition to supporting vegetative neurons, EGCs can also maintain intestinal mucosal integrity and homeostasis and regulate intestinal motility [7, 8]. Like astrocytes in the CNS, EGCs can express the activation marker glial fibrillary acidic protein (GFAP) and participate in the formation and maintenance of pain [8]. Recent studies have further shown that EGC function can be changed by many factors, such as pro-inflammatory cytokines, bacteria and neurotransmitters [9, 10]. After EGCs are activated, their phenotype changes, and this change is characterized by cell proliferation, morphological changes and the upregulation of GFAP.

Nerve growth factor (NGF) is widely distributed in the peripheral and central nervous systems. It plays an important regulatory role in the development, differentiation, growth, regeneration and functional characterization of peripheral and central neurons. In the gastrointestinal tract, many cells, such as gastrointestinal epithelial cells, EGCs, fibroblasts and other types of immune cells (mast cells, activated T lymphocytes), can secrete NGF and express its receptors [11]; thus, NGF participates in the regulation of gastrointestinal physiological function. NGF is also involved in the regulation of pain sensitivity [12, 13]. The intramuscular and subcutaneous injection of NGF in rats can cause rapid and delayed hyperalgesia [14, 15]. The intraperitoneal injection of NGF can also induce a reduction in the threshold of colonic pain, and there is a dose-effect relationship [16]. The expression of NGF in the colon during maternal deprivation (MD) is increased, and it is more sensitive to rectal distension. Treatment with an anti-NGF antibody during MD can inhibit the hypersensitivity reaction [17].

NGF and EGCs are associated with visceral hypersensitivity [17, 18] and gastrointestinal motility [19, 20]. In vitro, inflammatory cytokines can promote the secretion of NGF by EGCs and express the high affinity receptor tropomyosin receptor kinase A (TrkA) [11]. The expression of NGF in the intestinal mucosa of irritable bowel syndrome (IBS) patients is increased, and the phenotype of EGCs is changed [17]. However, it is not clear whether NGF/TrkA is expressed in the gastric mucosa of FD patients and whether the phenotype of EGCs is changed. The purpose of this study was to investigate the expression of NGF, its high affinity receptor TrkA and the EGC activation marker GFAP in the gastric mucosa in patients with FD and the association of these proteins with dyspeptic symptoms.

## Methods

### Study population

FD patients were diagnosed according to the Rome IV criteria. Patients that met the criteria were invited to participate in the study between 1 July 2016 and February 2018 at The Second Affiliated Hospital of Xi’an Jiaotong University, P. R. China. Patients with diseases of the gastrointestinal tract, liver, gallbladder, pancreas, or spleen, infectious colitis, helicobacter pylori infection, irritable bowel syndrome, gastrointestinal malignant disorders or endocrine diseases were excluded. Patients with psychiatric diseases were also excluded. Controls were selected from healthy subjects who underwent physical examination and patients that underwent gastroscopy for other indications (such as polyp review) but had a normal stomach. The characteristics of the subjects are shown in Table 1. The study was approved by the Ethics Committee of The Second Affiliated Hospital of Xi’an Jiaotong University, and all the subjects gave their written informed consent before participation. All subjects underwent gastroscopy, and the biopsy samples from the 27 FD patients and 13 controls were taken from the gastric antrum. Each subject yielded three biopsy samples. Two biopsies were fixed in buffered 10% formalin and then paraffin-embedded routinely, one for haematoxylin and eosin staining to exclude tumours, severe mucosal edema, or superficial erosion, another for immunocytochemistry and immunofluorescence. The third one was frozen in liquid nitrogen for protein extraction.

**Table 1:** Characteristics of the subjects

	Control	FD	EPS	PDS	EPS overlap PDS
Number	13	27	9	7	11
Gender(Male/female)	8/5	12/15	4/5	3/4	5/6
Age(years)	41.5 ± 11.3	42.1 ± 10.7	38.7 ± 13.1	46.1 ± 9.2	42.5 ± 9.3

According to the clinical symptoms of the FD patients and the inclusion criteria of the various subtypes, the FD patients were divided into three groups: the EPS group, the PDS group, and EPS overlap PDS. The upper gastrointestinal symptoms of the FD patients were evaluated according to the Rome IV criteria. The symptoms of the upper gastrointestinal tract were divided into epigastric pain, postprandial fullness, early satiation and epigastric burning. Scores of the severity of the symptoms were determined as follows: 0 points, no symptoms; 1 point, mild symptoms that can be felt; 2 points, moderate symptoms that do not affect daily life; 3 points, severe symptoms that affect daily working life.

### HE staining

Tissue was embedded in paraffin after fixation in 10% formalin and cut into 4 μm-thick sections. Then, the sections were stained with haematoxylin-eosin and photographed using an Olympus microscope. HE staining was mainly used to observe the morphology of gastric mucosal tissue and evaluate whether there was acute or chronic inflammation or a tumour.

### Immunohistochemistry

Embedded samples were deparaffinized, and antigen retrieved was performed. Sections were treated with 3% hydrogen peroxidase (30 min, room temperature) and then incubated with 5% donkey serum in phosphate-buffered saline (PBS) (60 min, room temperature). Then, the sections were incubated with rabbit anti-human NGF (1:200) (Abcam, Cambridge, UK) (overnight, 4 °C). PBS was used as a blank control. After washing with PBS, the sections were incubated with a goat anti-rabbit secondary antibody (Santa Cruz, Dallas, TX, USA) (60 min, room temperature). Specific reactivity was detected using a DAB kit (Zsbio, Beijing, China), and the sections were counterstained with haematoxylin. Images were captured at 400× under a light microscope (Olympus, Tokyo, Japan). The NGF expression was evaluated by the mean optical intensity under 5 fields using IPP 6.0 software (Media Cybernetics, Maryland, USA).

### Immunofluorescence

Embedded samples were deparaffinized, antigen retrieved was performed, and the sections were blocked. The sections were incubated with chicken an anti-human GFAP antibody (1:500) (Abcam, Cambridge, UK) and a rabbit anti-human TrkA antibody (1:150) (Abcam, Cambridge, UK) (overnight, 4 °C). PBS was used as a blank control. The cells were then incubated with a FITC-conjugated goat anti-chicken antibody and a rhodamine-conjugated goat anti-rabbit secondary antibody (60 min, room temperature) (Abbkine, California, USA). The nuclei were stained with DAPI (Sigma, St. Louis, USA). Images were captured at 400× under a fluorescent microscope (Olympus, Tokyo, Japan). The expression of GFAP and TrkA was evaluated by the average value of the ratio of the positive area to the visible area under 5 fields using IPP 6.0 software.

### Western blot analysis

Total protein was prepared using a protein extraction kit (Heart, Xi’an, China) according to the manufacturer’s protocol. Protein samples (50 μg) were loaded on polyacrylamide gels, and electrophoresis was carried out. Then, the proteins were transferred to PVDF membranes (Millipore Corp., Billerica, MA, USA). The PVDF membranes were blocked with 1× Tris-buffered saline-Tween (TBST) containing 5% non-fat milk (2 h, room temperature) and incubated with rabbit anti-human NGF, TrkA and β-actin in 1× TBS containing 5% skimmed milk (overnight, 4 °C). PBS was used as a blank control. After washing, the membranes were incubated with the appropriate secondary antibody (1 h, room temperature). The bands were visualized using a Super Signal Substrate Chemiluminescence kit and evaluated by IPP 6.0 software.

### Statistical analysis

SPSS 18.0 software (SPSS Inc., Chicago, IL) was used. The gender distribution was compared with the chi-square test. The measurement data in each group were firstly tested for normality using the Kolmogorov-Smirnov test. Age is presented as the mean ± standard deviation (SD) for normally distributed continuous variables, and the comparisons were determined by independent sample t-test and ANOVA. The expression of NGF, TrkA and GFAP are presented as the median and interquartile range for non-normally distributed variables, and a non-parametric Mann-Whitney test was used. Correlation analysis was performed by using the Spearman rank correlation coefficient. *P* < 0.05 or *P* < 0.01 was considered to be significant.

## Results

No significant differences in gender or age distribution were present among the controls compared the FD subgroups (Table 1).

### The protein expression of NGF in the gastric mucosa

**Fig. 1:**
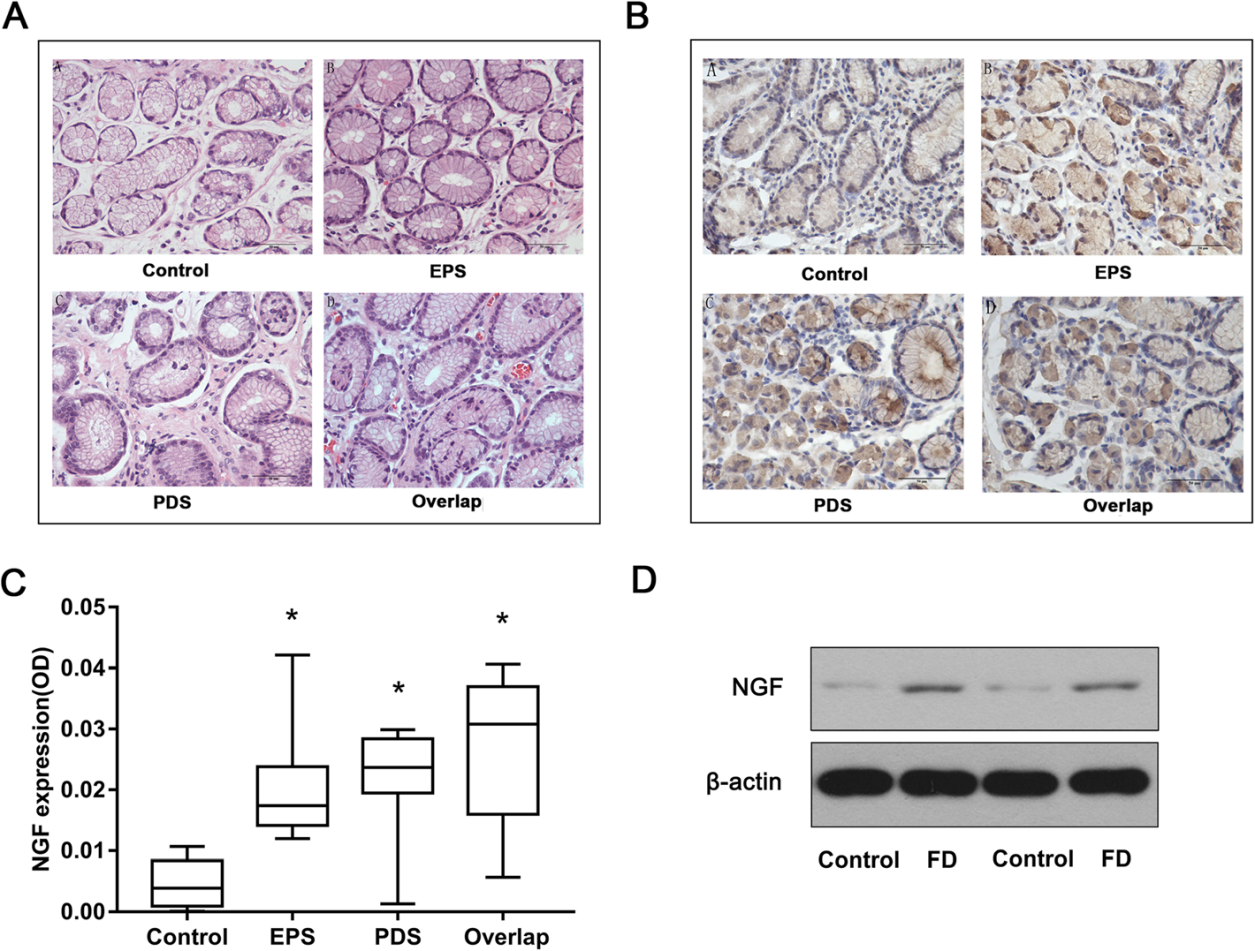
The protein expression of NGF in the gastric mucosa. **a**, HE staining of the gastric antrum in the FD subgroups and healthy control group (400×). **b**, **c**, The protein expression of NGF in the gastric mucosa was detected by immunohistochemistry (400×). *P < 0.01 compared with the control group. **d**, The protein expression of NGF in the gastric mucosa was detected by Western blot. β-actin served as a control

HE staining showed that there was no sign of acute inflammation in the antrum of FD and healthy control patients (Fig. 1a). Immunohistochemistry showed that NGF was positively stained as brownish yellow and expressed in the cytoplasm of the gastric epithelial cells, glandular epithelial cells, and mesenchymal cells of the lamina propria (Fig. 1b). The protein expression of NGF in the gastric mucosa of the patients in the FD subgroups was higher than that in the healthy controls (*P* < 0.05) (Fig. 1c). However, there was no significant difference among the subgroups (Fig. 1c). Western blot analysis indicated that the protein expression of NGF in the gastric mucosa of FD patients was higher than that in the healthy controls (Fig. 1d).

### The protein expression of TrkA in the gastric mucosa

**Fig. 2:**
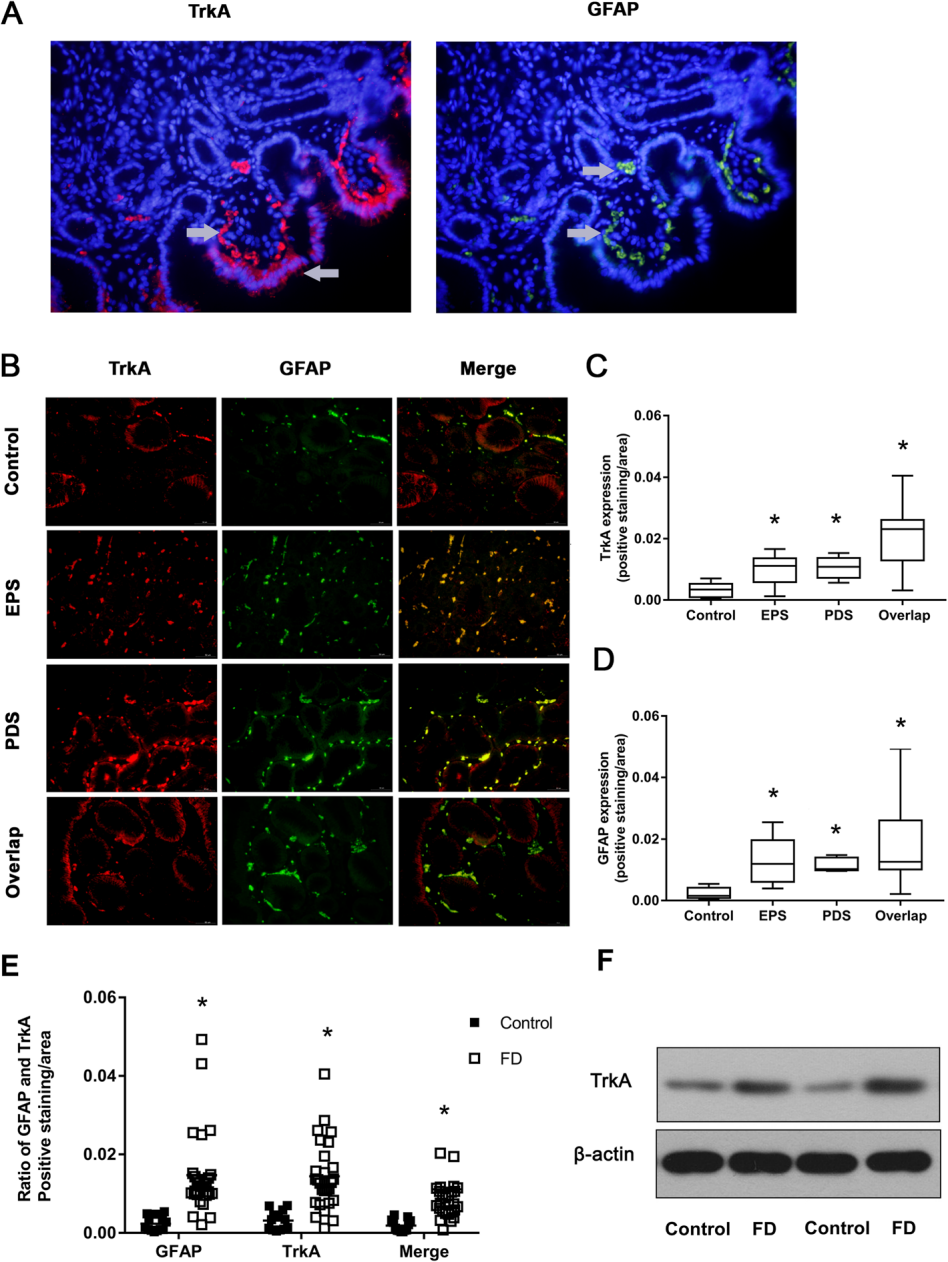
The protein expression of TrkA and GFAP in the gastric mucosa. **a**, Immunofluorescence for TrkA (red), GFAP (green) and DAPI (blue) (400×). **b**, The protein expression of GFAP and TrkA and their colocalization, as detected by double immunofluorescence (400×). **c**, The protein expression of TrkA in the gastric mucosa in the FD subgroups and healthy controls patients. *P < 0.01 compared with the control group. **d**, The protein expression of GFAP in the gastric mucosa. *P < 0.01 compared with the control group. **e**, The protein expression of TrkA in GFAP-positive EGCs. *P < 0.01 compared with the control group. **f**, The protein expression of TrkA in the gastric mucosa was detected by Western blot. β-actin served as a control

Immunofluorescence indicated that TrkA was positively stained as red fluorescence and observed in the cell membrane of some gastric epithelial cells and mesenchymal cells of the lamina propria (Fig. 2a, b). The protein expression of TrkA in the FD subgroups was higher than that in the healthy controls (*P* < 0.05). There was no significant difference among the FD subgroups (Fig. 2c). Western blot analysis indicated that the protein expression of TrkA in the gastric mucosa of FD was higher than that in the healthy controls (Fig. 2f).

### The protein expression of GFAP in the gastric mucosa

Immunofluorescence indicated that GFAP was positively stained as green fluorescence and expressed in the cells of the subepithelial lamina propria and periglandular areas in both FD patients and healthy controls (Fig. 2a, b). Double-staining experiments confirmed that TrkA was observed in EGCs, which exhibited GFAP-positive staining (Fig. 2b). The expression of GFAP in the gastric mucosa and in EGCs in patients in the FD subgroups was higher than that in the healthy controls (*P* < 0.05). There was no significant difference among the FD subgroups (Fig. 2d, e).

### The relationship between NGF, TrkA and GFAP expression and dyspeptic symptoms

**Fig. 3:**
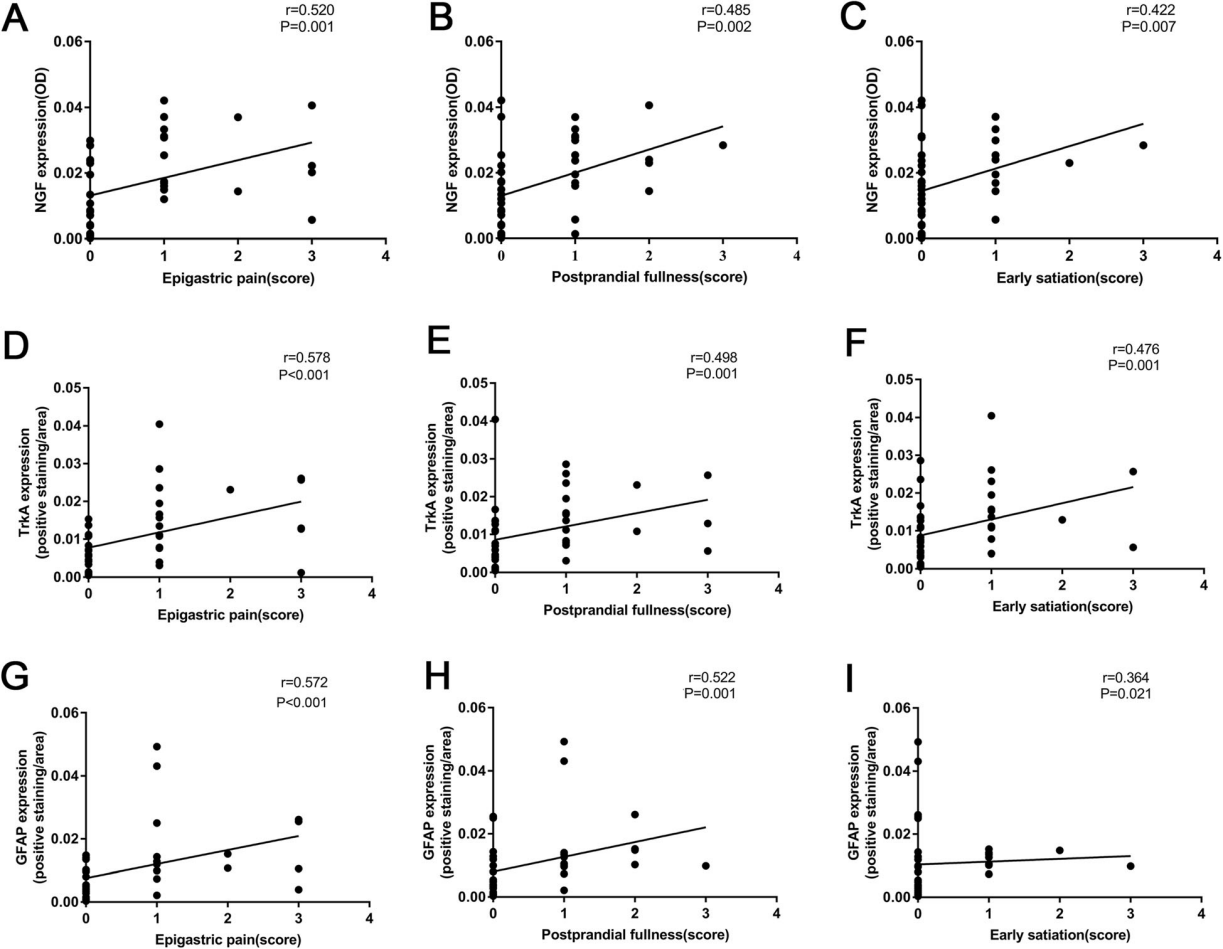
The relationship between NGF, TrkA and GFAP expression and dyspeptic symptoms. **a**, **b** and **c**, The relationship between the expression of NGF and epigastric pain, postprandial fullness, and early satiety in FD patients. **d**, **e** and **f**, The relationship between the expression of TrkA and epigastric pain, postprandial fullness and early satiety in FD patients. **g**, **h** and **i**, The relationship between the expression of GFAP and epigastric pain, postprandial fullness, and early satiety in FD patients

The expression levels of NGF, TrkA and GFAP were positively correlated with epigastric pain, postprandial fullness and early satiety in FD but were not related to epigastric burning (Fig. 3).

## Discussion

NGF, a major member of the neurotrophin family, functions through its high affinity receptor TrkA. In addition to being extensively distributed through the peripheral and central nervous systems, NGF has recently been found to also be distributed in peripheral tissues, and NGF/TrkA signalling pathways may be involved in the regulation of hyperalgesia [12]. IBS is a common functional gastrointestinal disease, and visceral hypersensitivity is one of its main pathogenic processes. NGF and TrkA are increased in the colorectal tissue of IBS patients [21, 22]. Barreau [17] found that the expression of NGF in the colons of MD rats is increased and is more sensitive to rectal dilatation stimulation. Treatment with an anti-NGF antibody can alleviate visceral hypersensitivity in rats. It has been suggested that NGF/TrkA may be involved in the occurrence of visceral hypersensitivity in IBS [23]. Visceral hypersensitivity is also one of the main pathogenic processes of FD, but the role of NGF/TrkA in the pathogenesis of FD has not yet been studied. Mertz [24] found that 87% of FD patients have functional changes in visceral afferent fibres, and thus experience reduced thresholds of pain and satiety, indicating that visceral hypersensitivity is the main pathological feature of FD and is the cause of abdominal distention and abdominal pain in FD patients. Winston [19] reported that the high expression of NGF in the stomach of chronic stress rats increases the visceral sensitivity of the stomach and that an NGF antibody can significantly inhibit the visceral hypersensitivity of the stomach. Bielefeldt [25] found that the expression of NGF in the gastric wall is increased in rats with gastritis and that it causes peripheral sensitization by acting on primary afferent neurons. Dothel [22] showed that TrkA is widely expressed on neuronal fibres both in the mucosa and within the myenteric and submucous plexus of the human colon. The mechanism by which the NGF-TrkA pathway modulates visceral hypersensitivity is complicated. It is accepted that NGF can evoke the excitability of sensory nociceptive fibres by altering the expression of sodium channels and increasing the expression of certain receptors, such as TRPV1, and key sensory neuropeptides, such as SP and CGRP, which are involved in the transmission of pain stimuli [23].

This study was the first to find that the expression levels of NGF and TrkA in the gastric mucosa of FD patients are significantly higher than those in the healthy controls and are positively correlated with the severity of postprandial fullness, early satiety, and epigastric pain; this suggests that the visceral hypersensitivity of FD may be related to NGF/TrkA.

Recently, studies have shown that acute or chronic stress [19, 22] and inflammation [11, 26] can increase the expression of NGF. In addition, EGCs, gastrointestinal epithelial cells, and some immune cells, such as mast cells and activated T lymphocytes, can secrete NGF [11, 27]. In vitro studies have also shown that inflammatory cytokines such as IL-1β and TNF-α can induce EGCs to secrete NGF and express its receptor TrkA [11]. Therefore, there are a number of factors that cause the increase of NGF expression in the gastric mucosa of FD patients observed in this study. First, this study showed that the expression of GFAP in the gastric mucosa of the patients with subtypes of FD was significantly higher than that in the control group, suggesting the activation of EGCs. Therefore, NGF in the gastric mucosa of FD patients may be synthesized and released by EGCs. Second, the gastric and duodenal mucosa of FD patients had a low degree of inflammatory infiltration, especially considering the increased number of mast cells and eosinophils [28]. Although this experiment did not detect the number of mast cells or other inflammatory cells in the gastric mucosa of FD patients, we found that NGF was expressed in some inflammatory cells and gastric epithelial cells in the lamina propria, which suggests that NGF may be secreted by inflammatory cells in the lamina propria and gastric gland epithelial cells.

EGCs, an important component of the ENS, are widely distributed throughout the gastrointestinal tract. Similar to that of astrocytes [29], the membrane of EGCs express a variety of receptors, such as the interleukin receptor, so that they can perceive changes in the environment and change their state. Bacterial invasion, inflammatory cytokines and neurotransmitter stimulation can cause EGC activation and the expression of GFAP [30]. It is believed that EGCs play a role in supporting and nourishing gastrointestinal neurons [31]. However, further research has shown that EGCs also regulate intestinal motility [8] and maintain intestinal mucosal integrity [7] and internal environmental homeostasis.

Cirillo [28] found that the submucosal plexus of the duodenum in FD patients exhibits glial hyperplasia and the expression of S100 in EGCs is increased. The present study showed that the expression of the EGC marker GFAP in the gastric mucosa in FD patients is significantly higher than that in healthy controls, which is consistent with Cirillo’s study. In addition, the present study also found that the expression of GFAP is positively correlated with the severity of postprandial fullness, early satiety, and epigastric pain, suggesting that EGCs may be associated with gastrointestinal motility and sensory abnormalities.

This study showed that TrkA, the receptor of NGF, is expressed on EGCs with immunofluorescence double staining. This relationship provides a basis for the interaction between EGCs and NGF. On the one hand, under the action of the inflammatory cytokines IL-1β and TNF-α, NGF secretion by EGCs is increased [11], and NGF participates in the disease by increasing the sensitivity of visceral sensations, regulating the permeability of the gastrointestinal mucosa epithelium, or inducing inflammatory reactions. On the other hand, NGF may be combined with the TrkA receptor on EGCs to further increase the expression of NGF, which can be considered a “positive feedback loop of autocrine mode.”

## Conclusion

This study suggests that the expression of NGF, TrkA and the EGC marker GFAP in the gastric mucosa of FD patients is increased, and these changes are associated with dyspeptic symptoms, which indicates that the interaction between NGF and EGCs may be involved in the pathogenesis of FD.

## Data Availability

The relevant raw data from this study can be readily available on request for non-commercial purpose per request from the corresponding author.

## Abbreviations

CNS: central nervous system
EGCs: enteric glial cells
ENS: enteric nervous system
EPS: epigastric pain syndrome
FD: Functional dyspepsia
GFAP: glial fibrillary acidic protein
IBD: irritable bowel syndrome
NGF: nerve growth factor
PBS: phosphate-buffered saline
PDS: postprandial distress syndrome
TrkA: tropomyosin receptor kinase A

## References

[1] Tack J, Talley NJ, Camilleri M, Holtmann G, Hu P, Malagelada JR, Stanghellini V (2006). Functional gastroduodenal disorders. Gastroenterology.

[2] Stanghellini V, Chan FK, Hasler WL, Malagelada JR, Suzuki H, Tack J, Talley NJ (2016). Gastroduodenal Disorders. Gastroenterology.

[3] Mahadeva S, Goh KL (2006). Epidemiology of functional dyspepsia: a global perspective. World J Gastroenterol.

[4] Shindo T, Futagami S, Hiratsuka T, Horie A, Hamamoto T, Ueki N, Kusunoki M, Miyake K, Gudis K, Tsukui T (2009). Comparison of gastric emptying and plasma ghrelin levels in patients with functional dyspepsia and non-erosive reflux disease. Digestion.

[5] Tack J, Caenepeel P, Fischler B, Piessevaux H, Janssens J (2001). Symptoms associated with hypersensitivity to gastric distention in functional dyspepsia. Gastroenterology.

[6] Furness JB (2012). The enteric nervous system and neurogastroenterology. Nat Rev Gastroenterol Hepatol.

[7] Snoek SA, Verstege MI, Boeckxstaens GE, van den Wijngaard RM, de Jonge WJ (2010). The enteric nervous system as a regulator of intestinal epithelial barrier function in health and disease. Expert Rev Gastroenterol Hepatol.

[8] Bassotti G, Villanacci V, Cathomas G, Maurer CA, Fisogni S, Cadei M, Baron L, Morelli A, Valloncini E, Salerni B (2006). Enteric neuropathology of the terminal ileum in patients with intractable slow-transit constipation. Hum Pathol.

[9] Bohorquez DV, Samsa LA, Roholt A, Medicetty S, Chandra R, Liddle RA (2014). An enteroendocrine cell-enteric glia connection revealed by 3D electron microscopy. PLoS One.

[10] Steinkamp M, Gundel H, Schulte N, Spaniol U, Pflueger C, Zizer E, von Boyen GB (2012). GDNF protects enteric glia from apoptosis: evidence for an autocrine loop. BMC Gastroenterol.

[11] von Boyen GB, Steinkamp M, Reinshagen M, Schafer KH, Adler G, Kirsch J (2006). Nerve growth factor secretion in cultured enteric glia cells is modulated by proinflammatory cytokines. J Neuroendocrinol.

[12] Li Q, Winston JH, Sarna SK (2016). Noninflammatory upregulation of nerve growth factor underlies gastric hypersensitivity induced by neonatal colon inflammation. Am J Physiol Regul Integr Comp Physiol.

[13] Apfel SC (2000). Neurotrophic factors and pain. Clin J Pain.

[14] Gerber RK, Nie H, Arendt-Nielsen L, Curatolo M, Graven-Nielsen T (2011). Local pain and spreading hyperalgesia induced by intramuscular injection of nerve growth factor are not reduced by local anesthesia of the muscle. Clin J Pain.

[15] Mills CD, Nguyen T, Tanga FY, Zhong C, Gauvin DM, Mikusa J, Gomez EJ, Salyers AK, Bannon AW (2013). Characterization of nerve growth factor-induced mechanical and thermal hypersensitivity in rats. Eur J Pain.

[16] Delafoy L, Raymond F, Doherty AM, Eschalier A, Diop L (2003). Role of nerve growth factor in the trinitrobenzene sulfonic acid-induced colonic hypersensitivity. Pain.

[17] Barreau F, Cartier C, Ferrier L, Fioramonti J, Bueno L (2004). Nerve growth factor mediates alterations of colonic sensitivity and mucosal barrier induced by neonatal stress in rats. Gastroenterology.

[18] Wang P, Du C, Chen FX, Li CQ, Yu YB, Han T, Akhtar S, Zuo XL, Tan XD, Li YQ (2016). BDNF contributes to IBS-like colonic hypersensitivity via activating the enteroglia-nerve unit. Sci Rep.

[19] Winston JH, Sarna SK (2013). Developmental origins of functional dyspepsia-like gastric hypersensitivity in rats. Gastroenterology.

[20] Qi R, Yang W, Chen J (2013). Role of enteric glial cells in gastric motility in diabetic rats at different stages. J Huazhong Univ Sci Technolog Med Sci.

[21] Willot S, Gauthier C, Patey N, Faure C (2012). Nerve growth factor content is increased in the rectal mucosa of children with diarrhea-predominant irritable bowel syndrome. Neurogastroenterol Motil.

[22] Dothel G, Barbaro MR, Boudin H, Vasina V, Cremon C, Gargano L, Bellacosa L, De Giorgio R, Le Berre-Scoul C, Aubert P (2015). Nerve fiber outgrowth is increased in the intestinal mucosa of patients with irritable bowel syndrome. Gastroenterology.

[23] Lewin GR, Lechner SG, Smith ES (2014). Nerve growth factor and nociception: from experimental embryology to new analgesic therapy. Handb Exp Pharmacol.

[24] Mertz H, Fullerton S, Naliboff B, Mayer EA (1998). Symptoms and visceral perception in severe functional and organic dyspepsia. Gut.

[25] Bielefeldt K, Ozaki N, Gebhart GF (2003). Role of nerve growth factor in modulation of gastric afferent neurons in the rat. Am J Physiol Gastrointest Liver Physiol.

[26] di Mola FF, Friess H, Zhu ZW, Koliopanos A, Bley T, Di Sebastiano P, Innocenti P, Zimmermann A, Buchler MW (2000). Nerve growth factor and Trk high affinity receptor (TrkA) gene expression in inflammatory bowel disease. Gut.

[27] Reinshagen M, Steinkamp M (2003). NGF---not just a nerve growth factor in the gut. Am J Physiol Regul Integr Comp Physiol.

[28] Cirillo C, Bessissow T, Desmet AS, Vanheel H, Tack J, Vanden Berghe P (2015). Evidence for neuronal and structural changes in submucous ganglia of patients with functional dyspepsia. Am J Gastroenterol.

[29] Ruhl A (2005). Glial cells in the gut. Neurogastroenterol Motil.

[30] Stoffels B, Hupa KJ, Snoek SA, van Bree S, Stein K, Schwandt T, Vilz TO, Lysson M, Veer CV, Kummer MP (2014). Postoperative ileus involves interleukin-1 receptor signaling in enteric glia. Gastroenterology.

[31] Yu YB, Li YQ (2014). Enteric glial cells and their role in the intestinal epithelial barrier. World J Gastroenterol.

